# Voluntary resistance wheel exercise from mid-life prevents sarcopenia and increases markers of mitochondrial function and autophagy in muscles of old male and female C57BL/6J mice

**DOI:** 10.1186/s13395-016-0117-3

**Published:** 2016-12-13

**Authors:** Zoe White, Jessica Terrill, Robert B. White, Christopher McMahon, Phillip Sheard, Miranda D. Grounds, Tea Shavlakadze

**Affiliations:** 1 School of Anatomy, Physiology and Human Biology, The University of Western Australia (UWA), 35 Stirling Highway, Crawley, WA 6009 Australia; 2 Centre for Cell Therapy and Regenerative Medicine, School of Medicine and Pharmacology, UWA and Harry Perkins Institute of Medical Research, Crawley, 6009 WA Australia; 3 School of Chemistry and Biochemistry, UWA, Crawley, 6009 WA Australia; 4 Developmental Biology Group, AgResearch Ltd, Hamilton, 3214 New Zealand; 5 Department of Physiology, University of Otago, Dunedin, 9010 New Zealand

**Keywords:** Aging, Muscle, Resistance exercise, Sarcopenia, Autophagy, Citrate synthase, Mitochondria, Oxidative capacity, Denervation

## Abstract

**Background:**

There is much interest in the capacity of resistance exercise to prevent the age-related loss of skeletal muscle mass and function, known as sarcopenia. This study investigates the molecular basis underlying the benefits of resistance exercise in aging C57BL/6J mice of both sexes.

**Results:**

This study is the first to demonstrate that long-term (34 weeks) voluntary resistance wheel exercise (RWE) initiated at middle age, from 15 months, prevents sarcopenia in selected hindlimb muscles and causes hypertrophy in soleus, by 23 months of age in both male and female C57BL/6J mice. Compared with 23-month-old sedentary (SED) controls, RWE (0–6 g of resistance) increased intramuscular mitochondrial density and oxidative capacity (measured by citrate synthase and NADH-TR) and increased LC3II/I ratios (a marker of autophagy) in exercised mice of both sexes. RWE also reduced mRNA expression of *Gadd45α* (males only) and *Runx1* (females only) but had no effect on other markers of denervation including *Chrng*, *Chrnd*, *Musk*, and *Myog*. RWE increased heart mass in all mice, with a more pronounced increase in females. Significant sex differences were also noted among SED mice, with *Murf1* mRNA levels increasing in male, but decreasing in old female mice between 15 and 23 months.

**Conclusions:**

Overall, long-term RWE initiated from 15 month of age significantly improved some markers of the mitochondrial and autophagosomal pathways and prevented age-related muscle wasting.

**Electronic supplementary material:**

The online version of this article (doi:10.1186/s13395-016-0117-3) contains supplementary material, which is available to authorized users.

## Background

Aging is associated with a loss of skeletal muscle mass and function, a condition known as sarcopenia [1–3]. In men and women, the annual rate of muscle mass loss is reported as approximately 0.9 and 0.7%, respectively, after the age of 75 years (reviewed in [4]). Sarcopenia can be greatly accelerated by physical inactivity and poor nutrition, and loss of function is more pronounced in the muscles of the lower limbs [5–7]. Sarcopenia can result in severe muscle weakness and contributes to frailty, reduced mobility, diminished independence, and an increased susceptibility to falls and fractures, with escalating costs to the global healthcare system [8]. Given the rapidly aging global population, research designed to better understand the molecular basis for the development, progression, and treatment of sarcopenia is of great importance [3].

Resistance exercise is an effective intervention used to counteract the detrimental effects of sarcopenia [9–12]. In humans aged >60 years, marked gains in strength, muscle mass (whole muscle and myofiber cross-sectional area (CSA)), functional mobility, muscle protein synthesis, and mitochondrial function have been observed after progressive resistance training programs that range from 8 weeks to 1 year [9, 10, 13–15]. These studies provide evidence that elderly men and women (including nonagenarians) are physiologically capable of adapting to progressive loading, and in some instances have reported relative gains in muscle strength and mass that are comparable to younger individuals [16, 17] and between sexes [14].

Voluntary wheel running (endurance or aerobic exercise) is often used to monitor the long-term benefits of exercise, with rodent models being widely used due to their relatively short lifespan; a 24-month-old mouse is considered roughly equivalent to a 70-year-old human [18]. Although an age-related decline in voluntary wheel running is well documented in mice and rats [19, 20], relatively small amounts of physical activity (≤1 km per day) can have many benefits [21–23]. Beyond the protection of muscle mass [19], long-term voluntary wheel running (ranging from 1 to 31 m) has a variety of physiological benefits including decreased weight gain [23, 24], restoration of neuromuscular junction (NMJ) architecture, and preserved muscle innervation [21, 25], increased mitochondrial biogenesis and autophagy [22, 26–28], improved oxygen uptake (VO_2_ max) [22], and the overall metabolic enhancement of the skeletal muscle [29].

Investigations in young men and elderly women (aged 22 to 75 years) show that combined resistance and endurance training can contribute to greater gains in muscle strength and/or mass, compared with endurance exercise alone [30–32]. Whether resistance exercise (with progressive loading of voluntary wheel running) can increase the hypertrophic potential of aging muscles has not been thoroughly tested in rodents.

We have previously shown in mice that short-term voluntary wheel running (10 weeks) combined with resistance is sufficient to induce hindlimb muscle hypertrophy in the quadriceps, gastrocnemius, and soleus (by up to 52%) of young male C57BL/6J mice (aged 16 weeks), and in the soleus (18%) of very old, sarcopenic mice (27 months) [24]. Little is known about the long-term adaptive molecular responses of the aging mouse skeletal muscle to voluntary resistance exercise and, in particular, whether this may differ between males and females.

The present study investigates the effect of 34 weeks of voluntary resistance wheel exercise (RWE) initiated from mid-life (15 months of age) on skeletal muscle mass and function in male and female C57BL/6J mice (aged 23 months). The effects of exercise, age, and sex on sarcopenia are described with respect to many aspects of mouse and muscle phenotype, as well as a range of molecular parameters, including mitochondrial density and oxidative metabolism, markers of protein degradation and protein synthesis, and the denervation of NMJs.

## Methods

### Mice and voluntary resistance wheel running protocol

Mature adult (14.5 months, male and female *N* = 24 of each sex) C57BL/6J mice were obtained from the Animal Resources Centre, Western Australia, and housed at the University of Western Australia under pathogen-free conditions. All experiments were conducted in accordance with guidelines of the National Health and Medical Research Council of Australia and were approved by the Animal Ethics Committee of the University of Western Australia.

Mice were maintained on a 12-h light-dark cycle (lights turned on at 07:00 h), at 22 ± 2 °C, with free access to a meat-free rat and mouse diet (protein, 20%; total fat, 4.8%; total fiber, 28.8%; total carbohydrate, 59.4%) fortified with vitamins and minerals (Specialty Feeds, Glen Forest, Western Australia) and drinking water. Mice were acclimated for 2 weeks prior to experimentation, and at 15 months of age were assigned to the following groups: (1) sedentary male (SED male; *N* = 10); (2) sedentary female (SED female; *N* = 9); (3) exercised male (EXE male; *N* = 6); (4) exercised female (EXE female; *N* = 7). The number of exercise chambers limited the number of EXE mice. A group of male (*N* = 8) and female (*N* = 8) C57BL/6J mice were also sampled at 15 months of age to obtain baseline data for healthy muscle prior to onset of the exercise.

Sedentary mice were housed individually in standard mouse cages with transparent walls (19.5 cm × 28 cm) for the duration of the experiment. Exercising mice were housed individually in Lafayette Mouse Activity Wheel Chambers (23.5 cm × 35.3 cm; Model 80820; Lafayette Instrument, IN, USA), equipped with a 12.7-cm diameter exercise wheel with a 5.72-cm wide running surface (Model 80820RW, Lafayette), and an adjustable servo-brake (Model 86070-B1) to control resistance application and wheel function. These wheels are considered to be low resistance or free-spinning wheels, as wheel inertia is very low (<1 g). Each chamber was equipped with an activity wheel counter (Model 86070A), to monitor wheel revolution, distance traveled (set at 0.40 m/revolution), and speed (m/min). The Activity Wheel Monitoring (AWM) software (Model 86065) was used to record all datasets. Wheel loading was determined by hanging known weights on each individual wheel and adjusting the brake to hold each selected weight as per manufacturer’s instructions [24]. These wheels were generously made available to us through an extended collaboration with Murray Goulburn Co-operative Co. Limited, Australia.

Voluntary resistance wheel exercise began at 15 months and lasted for 34 weeks, until mice reached 23 months. Exercising mice ran without resistance for the first 2 weeks (W1 and W2), with a 1 g increase in resistance every 2 weeks thereafter (up to 4 g; between W3 and W9). Mice were maintained at 4 g of resistance between W9 and W12 (4 weeks), before wheel loading was increased to 5 g between W13 and 20 (8 weeks) and 6 g between W21 and W34 (14 weeks). Values for distance run and speed were recorded every hour, for each mouse, throughout the duration of the study using AWM software, and the averages shown were taken over 3–5 days. Body weights were recorded for each mouse three times a week, and food consumption was recorded weekly for the duration of the experiment. Data were individually corrected for body weight and presented as an amount consumed per day. Wheels were locked 3–4 h prior to sampling (starting from 0700 h) to avoid any acute effects of wheel running.

### External work

External work was calculated using the equation in [33]. To calculate average daily work, the torque necessary to maintain wheel speed at a given load was calculated as *τ* = *mg* × *r*, where *τ* is torque, *m* is wheel load, *g* is Newton’s conversion factor (9.81 m/s^2^), and *r* is radius of the cage wheel. Work (*W*) was calculated as *W* = *τ* × *θ*, where *θ* is angular displacement (2π radians/revolution and, therefore, 15, 750 radians for 1 km). External work was adjusted for each individual animal body weight (in kg). Thus, for each animal: *W* = *τ* × 15.75 × distance run (km)/mouse mass (kg).

### Tissue collection

Mice were killed by cervical dislocation while under terminal anesthesia (2% *v*/*v* Attane isoflurane, Bomac, NSW, Australia, 400 mL NO_2_ and 1.5 L O_2_). Muscles from the hind and forelimbs, including the quadriceps femoris, gastrocnemius, tibialis anterior (TA), soleus, extensor digitorum longus (EDL), and triceps brachii, were excised, weighed, and snap frozen in liquid nitrogen. In this study, only the quadriceps muscles were used for protein and mRNA isolation, while quadriceps and gastrocnemius muscles were both used to examine citrate synthase activity. Soleus and quadriceps muscles were cut transversely in the middle, mounted onto tragacanth gum (Sigma-Aldrich Pty Ltd, Sydney, Australia) and frozen in liquid nitrogen cooled isopentane for histological analyses. Epididymal fat pads were weighed, and the length of the tibial bones was measured and used for normalizing wet muscle weights.

### Protein extraction and immunoblotting

Protein was extracted from the quadriceps muscles as detailed elsewhere [34]. The muscles were ground in liquid nitrogen, and the powder homogenized in ice-cold PBS, 1% NP40, 1 mM EDTA buffer, supplemented with complete EDTA-free protease inhibitor and PhosSTOP phosphatase inhibitor tablets (Roche, Manheim, Germany), and centrifuged at 13,000*g* for 20 min at 4 °C. The supernatant represents the 1% NP40 soluble protein fraction. Resultant pellets were resuspended in a buffer containing 20 mM HEPES (pH 7.5) and 4% SDS, supplemented with protease and phosphatase inhibitor tablets (Roche, Manheim, Germany), and solubilized by sonication 4 × 5 s bursts at 40% amplitude (Vibra Cell, Sonics & Materials Inc. #VCX 130), followed by centrifugation at 19,600*g* for 10 min at 16 °C [34].

Protein was quantified with a DC™ protein assay (Bio-Rad, NSW, Australia). Samples were resolved on 4–15% SDS-PAGE TGX gels (Bio-Rad, NSW, Australia, #456-1086) and transferred onto nitrocellulose membranes (Bio-Rad, NSW, Australia, #170-4158), using a Trans Turbo Blot system (Bio-Rad, NSW, Australia). Immunoblotting was performed with antibodies to p-AKT(Ser473) (#9271), t-AKT (#9272), p-ribosomal protein S6(Ser235/236) (#4858), t-ribosomal protein S6 (#2217), p-S6K1 Kinase(Thr389) (#9205) (detects p70S6K (S6k1) and p85S6K), t-S6K1 Kinase (#9202), p-ULK1(Ser757) (#6888), t-ULK1 (#8054), LC3B (#2775), SQSTM1/p62 (#5114), and GAPDH (#2118) all from cell signaling (all 1:1000 in 5% BSA). The “p” and “t” prefixes signify “phosphorylated” and “total” forms, respectively. HRP-conjugated secondary antibodies were from Thermo Fisher Scientific, MA, USA. Chemiluminescence signal was captured using the ChemiDoc MP Imaging System (Bio-Rad, NSW, Australia), and digital images were generated. Resultant images were converted into a TIFF format and quantified using ImageJ software. A common sample was loaded onto each gel to normalize for detection efficiencies across membranes. Proteins that were immunoblotted on the same membrane share an image with GAPDH as the loading control.

### RNA extraction and quantitative real-time PCR (RT-qPCR)

RNA was extracted using the RNeasy® Fibrous Tissue Mini Kit (Qiagen VIC, Australia, #74704), and 1 μg was reverse transcribed using a QuantiTect Reverse Transcription Kit (Qiagen, VIC, Australia, #205311). QuantiTect primers were all purchased from Qiagen: (Muscle RING finger protein-1 (*Murf1*) #QT00291991; muscle atrophy F-box protein 32 (*Atrogin-1*) #QT00158543); growth arrest and DNA damage-inducible 45 α (Gadd45α) #QT00249655; runt-related transcription factor-1 *(Runx1)* #QT00100380; nicotinic acetylcholine receptor δ subunit *(Chrnd)* #QT00199472; nicotinic acetylcholine receptor γ subunit (*Chrng*) #QT00100268; muscle, skeletal, receptor tyrosine kinase *(Musk) #*QT00197792, and Myogenin *(Myog) #*QT00112378*.* RT-qPCR was performed using SYBR green chemistry (GoTaq qPCR Master Mix; Promega) on a Rotorgene-Q qPCR thermal cycler (Qiagen, VIC, Australia). Gene expression in quadriceps muscles was normalized to the geometric mean of *Hprt* and *Ppia* expression values (hypoxanthine phosphoribosyl-transferase 1 (*Hprt1*) *#*QT00166768; peptidylprolyl isomerase A (*Ppia*) #QT00247709) [35]. The mRNA levels of these genes were similar across all ages and between SED and EXE cohorts. Data are expressed as mean ± SEM from *N* = 5–10 biological replicates.

### Citrate synthase (CS) activity to measure mitochondrial density

CS activity was measured in both quadriceps and gastrocnemius muscles as described elsewhere [36]. In brief, the snap frozen muscles were ground in liquid nitrogen and homogenized in a buffer containing 5 mM HEPES (pH 8), 1 mM EGTA, 5 mM MgCl_2_, 1 mM dithiothreitol, and 0.1% TritonX-100. After centrifugation, protein concentrations were measured using the DC protein assay (Bio-Rad, NSW, Australia), and all samples were frozen at −80 °C until analysis. Samples were defrosted and aliquoted into a 96-well plate. Tris buffer (0.1 M, pH 8) was added to each well, and both a 5,5-dithiobis-(2-nitrobenzoic acid) (DTNB) solution and acetyl CoA solution added and the plate vortexed. A solution containing oxaloacetate was added to initiate the reaction and assayed immediately; absorbance was recorded at 412 nm every 30 s for 5 min. The protein concentration of each sample was again measured using the DC protein assay, to give a measurement of nmol min^−1^ mg protein^−1^.

### Hematoxylin and eosin (H&E) and nicotinamide adenine dinucleotide nitro-blue tetrazolium (NADH-TR) staining

Transverse frozen sections (8 μm) of the soleus muscle were stained with H&E to assess general tissue architecture, and to quantify myofiber number and percentage of myofibers with centralized nuclei using a standard protocol. The quadriceps muscles stained with NADH-TR were used to quantify changes in the oxidative state of whole muscle sections, which can be used as a complementary measure of mitochondrial density [37, 38].

Briefly, a 1:1 mixture of NBT (nitro-blue tetrazolium) solution (2 mg/mL; NBT/0.05 M TRIS pH 7.6) (N6876, Sigma-Aldrich Pty Ltd, Sydney, Australia) and NADH (nicotinamide adenine dinucleotide, reduced) solution (1.6 mg/mL; NADH/0.05 M TRIS pH 7.6) was added to muscle sections and incubated for 30 min at 37 °C. Incubated sections were washed with three exchanges of tap water, and unbound NBT removed with three exchanges of 30, 60, and 90% acetone-deionized water in increasing, then decreasing concentration. Sections were left in 90% acetone until a faint purple cloud could be seen over the section; then sections were rinsed with three exchanges of tap water and mounted with DPX mountant for microscopy (06522, Sigma-Aldrich Pty Ltd, Sydney, Australia), air-dried, and stored at room temperature.

### Laminin immunostaining

Transverse frozen sections (8 μm) of the soleus muscle were stained with polyclonal rabbit anti-PAN laminin antibody (L9393, Sigma, Australia; dilution 1:300) (which labels myofiber basement membranes). The primary antibody was detected by goat anti-rabbit Alexa594 antibody (A-11012, Invitrogen, Molecular Probes, OR, USA; dilution 1:500). Laminin-stained sections were used to measure myofiber CSA and determine myofiber size distribution.

### Image acquisition and morphometric analysis

The soleus was selected for detailed analysis because it showed the most pronounced hypertrophic response to exercise in both male and female mice. Tiled images of the soleus muscles stained with H&E, and laminin antibodies were captured at ×20 magnification using a Nikon Eclipse Ti inverter microscope equipped with Nikon DS-Fi2 camera (Nikon Corporation, Tokyo, Japan) for bright field imaging, and CoolSNAP EZ camera (Roper Scientific Photometrics, Ottobrunn, Germany) for fluorescence imaging. Images were captured using NIS-Elements BR 4.1 software, and analyses performed using ImagePro Plus 4.5 (Media Cybernetics, MD, USA) and FoveaPro software (Reindeer Graphics, Asheville, NC, USA).

The quadriceps muscle was also selected for further analysis because the loss of muscle mass was prevented by exercise and atrophied similarly with age in both male and female mice. Whole quadriceps muscle cross sections stained with NADH-TR were scanned using a Leica Aperio ScanScope XT digital slide scanner (Leica Biosystems, Wetzlar, Germany). An average of 2 to 5 transverse sections for each quadriceps muscle was used to estimate the mean (or total) intensity of NADH-TR staining, as well as the proportion of each section occupied by weak, intermediate, or strong NADH-TR staining. Intensity measures were made using Aperio ImageScope software (Leica Biosystems).

All non-tiled images of transverse muscle sections were captured at ×20 magnification using a Nikon 90i microscope equipped with Nikon DS-Fi2 camera. Images were captured using NIS-Elements AR 3.0 software (Laboratory Imaging Ltd., Czechoslovakia).

### Statistical analysis

Longitudinal data were subjected to repeat measures analysis of variance (ANOVA) using GenStat 17th Edition with the five treatment groups included in the treatment structure statement. Post hoc multiple comparisons were performed using the Student-Newman-Kuels method where single comparisons between means were made, or the method of Tukey where multiple comparisons of the same means were used [39].

Data comparisons between age and sex and sex and activity (between SED and EXE) were analyzed by two-way ANOVA, with a significance threshold of *P* ≤ 0.05 for direct mean comparisons. Where an interaction between both factors was detected, independent sample *t* tests were used to derive differences, and these *P* values are indicated in the “Results” section. Data are presented as means with the standard error of the mean (SEM).

## Results

### Body weights

**Fig. 1:**
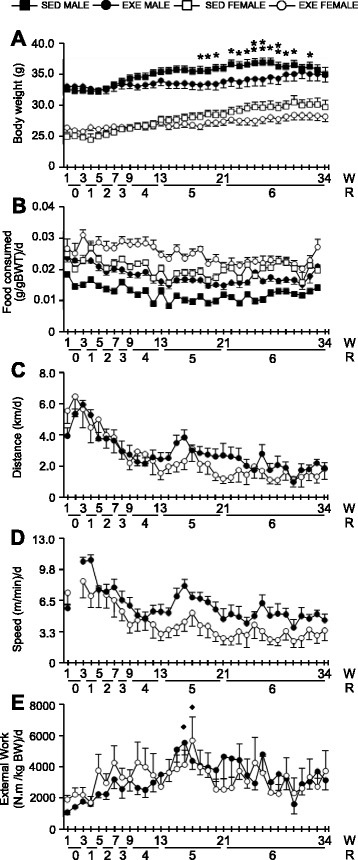
Average weekly body weights (**a**), food consumption (**b**), running distance (**c**), speed (**d**), and external work (**e**) in males and females in both SED and RWE cohorts over 34 weeks. The *asterisks* indicate significant differences between sedentary and exercised mice (data were pooled for sex; *P < 0.05; **P < 0.01). Diamond indicates a significant effect of age ^♦^ *P* < 0.05. Data for “speed” at week 2 are missing due to technical issues. For each weekly time point, data are mean ± SEM. *N* = 6–10 mice per group

Male mice were heavier than females (*P* < 0.001), and body weight increased throughout the study in SED mice of both sexes (*P* ≤ 0.05). By contrast, exercise prevented body weight gain and this became more apparent with wheel loadings of 5 and 6 g (*P* < 0.001) (Fig. 1a).

### Food consumption (normalized to body weight)

Females consumed 24% more food than males overall (*P* < 0.001): SED females ate 28% more than SED males (*P* < 0.001) and exercised females ate 19% more than exercised males (*P* < 0.001), (Fig. 1b). Overall, exercising mice ate consistently more than SED mice for both sexes (*P* < 0.001).

### Running distance and speed

The distance and speed that mice ran increased from week 1 in males to peak at week 3 (*P* < 0.05). The pattern was similar for females over the first 3 weeks, and there was no significant difference between sexes. Following the peak, the distance and speed that mice ran declined over the remainder of the study (*P* < 0.001), with no differences between sexes (Fig. 1c, d).

### Rate of external work

The rate of external work was the same for both sexes and increased (*P* < 0.01) in both sexes (threefold in females and fivefold in males) to peak between weeks 15 and 17 (Fig. 1e). Although work declined in the later part of the study, the levels of work expended remained equal to week 1 levels.

### Pattern of running activity

**Fig. 2:**
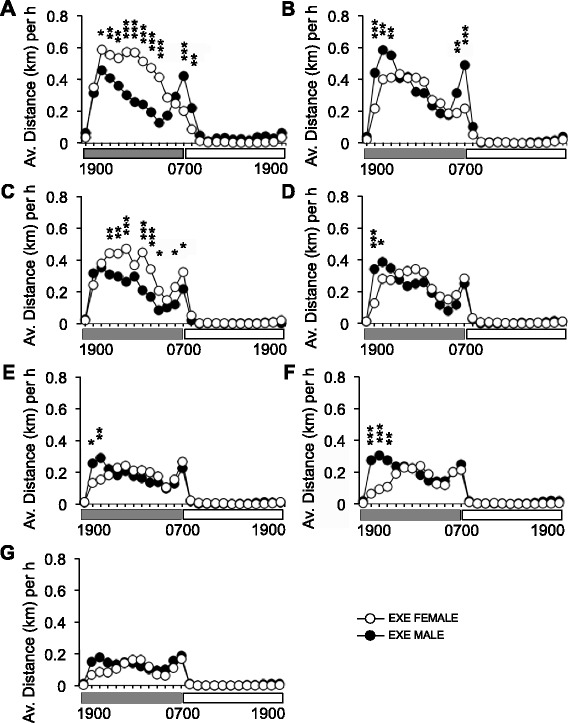
Average distances run by male and female mice per hour over 24 h, over 34 weeks of RWE. Graphs (**a**–**g**) depict average running distances by male and female mice at each level of resistance (0–6 g) throughout the exercise protocol: weeks 1 and 2 with resistance set at 0 g (**a**); weeks 3 and 4 (1 g) (**b**); weeks 5 and 6 (2 g) (**c**); weeks 7 and 8 (3 g) (**d**); weeks 9 to 12 (4 g) (**e**); weeks 13 to 20 (5 g) (**f**); and weeks 21 to 34 (6 g) (**g**). *N* = 6–7 mice per group. *Gray* and *white bars* under the X-axis indicate scotophase (1900–0700 h) and photophase (0700–1900 h). *Error bars* were omitted for clarity. *Asterisk* denotes a significance between males and females; *P < 0.05; **P < 0.01; ***P < 0.001

There was a clear pattern of running behavior, where both sexes ran during scotophase (lights out) but not during photophase (lights on) (*P* < 0.001). There were two major peaks of running activity. The first occurred during the initial 2 h of scotophase (Fig. 2a–g), and the second period of activity was initiated 2 h before the onset of photophase and peaked when lights were switched on. There was also a clear decline in the distances run as loads increased, for both sexes (*P* < 0.001). Females ran more during scotophase when loads were 0 and 2 g, whereas males tended (*P* = 0.053) to run more than females during the first 3 h of scotophase with loads from 4 to 6 g (Fig. 2a–g). The second running peak at the end of scotophase is still evident at the end of the exercise regime at 23 months, even though all running is greatly reduced at this stage.

### Impact of age on muscle mass and body composition for sedentary (SED) mice

The incidence of sarcopenia was greater in the hindlimb muscles of SED male compared with female mice between 15 and 23 months of age. Both forelimb and hindlimb muscle weights were standardized to tibial bone length. The standardized mass of the quadriceps, gastrocnemius, soleus, and TA decreased by 15, 11, 18, and 14%, respectively, in SED males (*P* < 0.05; Table 1), while in female mice, a 13% decrease in quadriceps muscle mass was observed (with no significant changes to other muscles) (*P* < 0.05; Table 1). By contrast, standardized weights of the triceps brachii were unaffected by age in either sex (Table 1). Overall, males had consistently higher muscle weights compared with females at both 15 and 23 months of age (*P* < 0.001), except in the soleus where the standardized mass was similar between SED mice at 23 months (Table 1).

**Table 1:** Phenotypic characterization and muscle weights standardized to tibia length for male and female C57BL/6J mice at middle age (15-month SED), old age (23-month SED), and following 34 weeks of RWE intervention (23-month EXE)

	Male			Female		
	15-month SED	23-month SED	23-month EXE	15-month SED	23-month SED	23-month EXE
Phenotype characteristics						
Abdominal fat pad (% BW)	1.0 ± 0.3	2.8 ± 0.4*	1.8 ± 0.3	0.8 ± 0.1	2.0 ± 0.4*	1.3 ± 0.2
Blood glucose (mmol/L)	10.7 ± 0.3	9.2 ± 0.4	8.7 ± 0.5	11.6 ± 1.0	9.9 ± 0.4*	9.6 ± 0.3
Tibia length (cm)	1.80 ± 0.01	1.80 ± 0.01	1.82 ± 0.2	1.82 ± 0.01	1.81 ± 0.01	1.83 ± 0.01
Heart weight (mg)	174.6 ± 7.2°	165.3 ± 6.2°	187.3 ± 7.0°^♦^	135.4 ± 7.1	145.0 ± 5.0	169.3 ± 7.0^♦^
Whole muscle weights						
Quadriceps (g)/tibia (cm)	119.1 ± 3.6°°°	101.6 ± 2.6*°°°	112.4 ± 2.9°^♦^	87.7 ± 2.3	76.7 ± 2.0*	84.6 ± 1.6^♦^
Gastrocnemius (g)/tibia (cm)	93.8 ± 3.3°°°	83.1 ± 2.0*°°°	89.2 ± 2.7°^♦^	67.4 ± 1.3	66.2 ± 2.3	69.9 ± 1.5
Soleus (g)/tibia (cm)	5.5 ± 0.3 °	4.5 ± 0.2*	6.6 ± 0.3^♦♦♦^	4.7 ± 0.2	4.5 ± 0.2	5.7 ± 0.4^♦♦♦^
TA (g)/tibia (cm)	28.0 ± 1.7°°°	24.1 ± 0.7*°°°	26.5 ± 1.7°	20.5 ± 1.0	18.3 ± 0.3	20.1 ± 1.2
EDL (g)/tibia (cm)	6.8 ± 0.4°°°	6.3 ± 0.3°°°	7.3 ± 0.1°^♦^	5.6 ± 0.2	4.9 ± 0.2	5.9 ± 0.5^♦^
Triceps (g)/tibia (cm)	72.6 ± 2.2°°°	68.2 ± 1.2°°°	68.5 ± 2.5°	52.2 ± 1.0	52.0 ± 2.3	53.2 ± 1.2

Values are means ± SEM

*EDL* extensor digitorum longus, *EXE* exercise, *NS* no significance, *RWE* resistance wheel exercise, *SED* sedentary, *TA* tibialis anterior

*Significant difference between 15-month SED within the same sex; *P < 0.05

^♦^Significant difference between 23-month SED within the same sex; ^♦^ *P* < 0.05; ^♦♦^ *P* < 0.05; ^♦♦♦^ *P* < 0.05

°Significance between male and female mice in the same group; °*P* < 0.05; °°*P* < 0.05; °°°*P* < 0.05

When expressed as a percentage of body weight, abdominal fat mass increased with age by 181 and 152% in SED males and females, respectively (*P* < 0.001; Table 1), with no sex specific differences observed. In females, aging was also accompanied by a 15% decrease in blood glucose levels (*P* < 0.05; Table 1); although a comparable age-related decrease was observed in male mice (13.7%), statistical significance was not reached. Age had no effect on heart weight in either male or female mice; however, heart weight was higher in SED males relative to females at both 15 and 23 months of age (*P* < 0.05; Table 1). No age-related or sex-specific differences to the tibia length were observed.

### Impact of voluntary resistance wheel exercise (RWE) on muscle mass and body composition

In male mice, voluntary RWE initiated from middle age (15 months) prevented the age-related decline in mass seen for the SED quadriceps, gastrocnemius, and EDL muscles, with retention of 11, 10, and 16%, respectively, of muscle mass at 23 months: muscle mass of exercised old mice was comparable to healthy baseline controls (15 months), (*P* < 0.03; Table 1). A similar maintenance of muscle mass (prevention of sarcopenia) was observed in exercised female mice, but was significant only for the quadriceps and EDL muscles (10 and 19%, respectively; *P* < 0.04; Table 1). The soleus muscle exhibited the greatest hypertrophic response to RWE, with the standardized mass in both sexes exceeding baseline control weights (15 months) by approximately 22% (*P* < 0.001; Table 1).

Overall in exercised mice (aged 23 months), abdominal fat mass measured as a percentage of total body weight, was reduced by approximately 34% (with no sex specific differences observed) (*P* < 0.03; Table 1). In response to RWE, female mice (aged 23 months) had greater cardiac hypertrophy (25%) than males (7.3%), relative to 23-month-old SED mice (*P* < 0.05; Table 1). Blood glucose levels were unaffected by RWE, and no effects on the tibia length were observed between middle-aged and old cohorts (Table 1).

### Myofiber CSA, number, and central nucleation

**Fig. 3:**
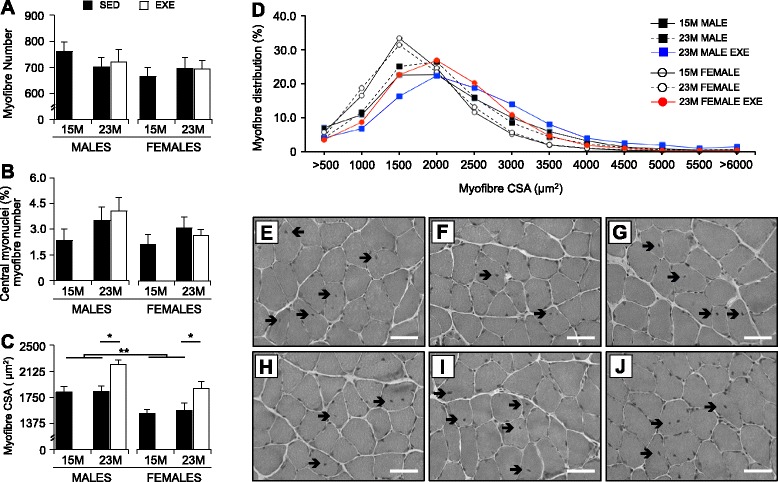
Morphometric characterization of the soleus muscles from 15-months SED, 23-month SED, and 23-month RWE mice, of both sexes. Entire transverse sections of the soleus muscles stained with H&E were used to quantify the number of individual muscle fibers (**a**) and proportion of myofibers with non-peripheral nuclei or central nucleation (**b**). Average myofiber size (as measured by cross-sectional area (CSA)) (**c**) and myofiber size distribution (**d**) were quantified on transverse section of the soleus immunostained for laminin. Myofibers with displaced or central nuclei were seen in the soleus muscles of male (**e**–**g**) and female (**h**–**j**) mice at both 15-month SED (**e**, **h**) and 23-month SED (**f**, **i**), as well as after RWE (**g**, **j**) (*arrows*). Data were analyzed by ANOVA, using age and sex and sex and activity as variables. Data are mean ± SEM. *Asterisk* denotes significance at *P < 0.05; **P < 0.01; ***P < 0.001. For each age group, *N* = 6–10 mice/group. Scale is 50 μm

Transverse sections of soleus stained with H&E were used to assess general muscle morphology and to quantify myofiber number and percentage of central nuclei (Fig. 3a, b). The size and percentage distribution of individual myofibers was quantified on transverse sections of the soleus immunostained with laminin, which defines myofiber contours (Fig. 3c, d). The soleus was selected for detailed histological analyses because of the pronounced hypertrophic response to RWE in both sexes.

In SED mice, the number and size (CSA) of myofibers in the soleus of both sexes were similar between 15 and 23 months of age (Fig. 3a, c), although the female soleus muscles were smaller overall in CSA compared with male soleus (*P* = 0.002; Fig. 3c). Central nucleation of myofibers was present in the soleus muscles of both male (Fig. 3e–g) and female (Fig. 3h–j) mice at 15 months (Fig. 3e, h) and 23 months of age (Fig. 3f, i).

Exercise had no significant effect on myofiber number (Fig. 3a) or the percentage of central nucleation in the male and female soleus muscles (Fig. 3b, g, j), although exercise increased myofiber CSA relative to SED controls by 21% in males and 20% in females (*P* < 0.05; Fig. 3c). Further analysis of myofiber CSA distribution showed that old male and female mice (at 23 months) had myofiber profiles clustered at 1500–2000 μm and 1500 μm, respectively (Fig. 3d), whereas after RWE, more myofibers were clustered between 2500 and 3500 μm in males and 2000–3500 μm in females (Fig. 3d).

### Citrate synthase (CS) activity as a measure of mitochondrial density

**Fig. 4:**
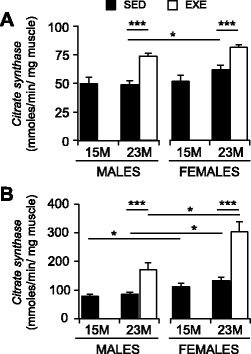
Citrate synthase activity in the quadriceps and gastrocnemius muscles of 15-month SED, 23-month SED, and 23-month RWE mice, of both sexes. Citrate synthase activity in the quadriceps (**a**) and gastrocnemius (**b**) muscle lysates was used as a marker of mitochondrial density. Data were analyzed by ANOVA, using age and sex and sex and activity as variables. Data are mean ± SEM. *Asterisk* denotes significance at *P < 0.05; **P < 0.01; ***P < 0.001. For each age group, *N* = 6–10 mice/group

Citrate synthase (CS) is a mitochondrial enzyme used as a marker of mitochondrial density in tissues [38, 40]. The CS activity was measured in the quadriceps and gastrocnemius muscles of male and female mice (Fig. 4a, b). In SED mice, both muscles showed similar levels of CS activity at 15 and 23 months of age (irrespective of sex), although CS activity in the quadriceps was maintained at higher levels in 23-month-old females compared with males (*P* < 0.05; Fig. 4a), and CS activity in the gastrocnemius was higher in SED females than males at both 15 and 23 months (Fig. 4b; *P* < 0.001 main effect).

In the quadriceps, exercise increased CS activity by 52% in males and 32% in females, which exceeded both middle-aged (15 months) and old (23 months) SED levels (*P* < 0.001; Fig. 4a). In the gastrocnemius, exercise also increased CS activity in old males (101% increase) and females, with a greater response in females (128% increase) (Fig. 4b; *P* < 0.001).

### NADH-TR staining of quadriceps as measure of oxidative metabolism

**Fig. 5:**
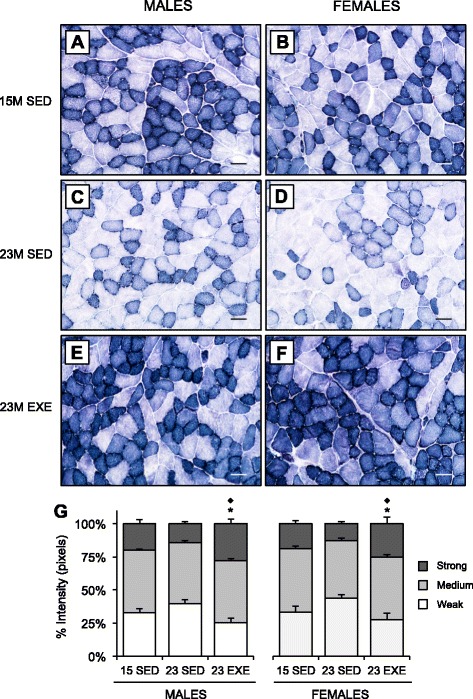
NADH-TR staining intensity in the quadriceps muscles of 15-month SED, 23-month SED, and 23-month RWE mice, of both sexes. Transverse sections of the quadriceps muscles stained with NADH-TR were used to quantify changes to oxidative state. Representative images were taken at ×20 magnification in male (**a**, **c**, **e**) and female (**b**, **d**, **f**) mice from both 15-month SED (**a**, **b**) and 23-month SED (**c**, **d**) cohorts, as well as after RWE (**e**, **f**) in the rectus femoris. Scale is 50 μm. NADH-TR intensity was quantified by calculating the percentage of weak, intermediate, or strong staining intensity of the whole muscle relative to total pixel intensity (**g**). Data for each intensity level was analyzed by ANOVA, using age and sex and sex and activity as variables. *Asterisks* indicate a significant decrease in the percentage of weakly staining intensity and *diamond* an increase in the percentage of strongly staining intensity between 23-month SED and 23-month EXE cohorts of the same sex (*P* < 0.05). Data are mean ± SEM. For each age group, *N* = 6–9 mice/group

Transverse sections of the quadriceps were stained with NADH-TR to analyze the oxidative profile of whole muscle sections, which can be related to mitochondrial density [37, 38]. The quadriceps muscles of SED 23-month-old mice (both males and females when considered together) had reduced NADH-TR staining intensities relative to 15 months, with an increase in the overall percentage of weak staining present in muscle sections (30% increase in weak staining relative to strong staining; main effect, *P* = 0.01). This, however, was not significant upon individual analysis of SED males or females between 15 and 23 months. A corresponding decrease in the overall percentage of strong staining intensity was also noted (main effect, *P* = 0.007; Fig. 5 compared a, b to c, d and Fig. 5g), although again this was not significant upon individual analysis of SED males or females between 15 and 23 months of age. Intermediate staining was unaffected by age or gender (Fig. 5g).

Exercise prevented age-related changes to muscle oxidative profiles, with a striking increase in the percentage of stronger staining intensities observed after RWE in both sexes; 94 and 95% in males and females, respectively, compared with SED 23 months controls (*P* < 0.001; Fig. 5 compared c, d to e, f; Fig. 5g). This occurred concomitantly with a reduction in the percentage of weak staining intensities (Fig. 5g).

### Analyses of ULK1, LC3, and p62 as measures of autophagy

Since mTORC1 inhibits autophagy via phosphorylation of Unc51-like kinase 1 (ULK1) [41–44], phosphorylation of ULK1 on the mTORC1-specific residue (Ser757) was quantified. In addition to ULK1 phosphorylation, we measured protein levels of LC3 (microtubule-associated protein light chain 3) and p62, also known as sequestosome 1 (SQSTM1), as these are used as autophagy markers [45–47].

**Fig. 6:**
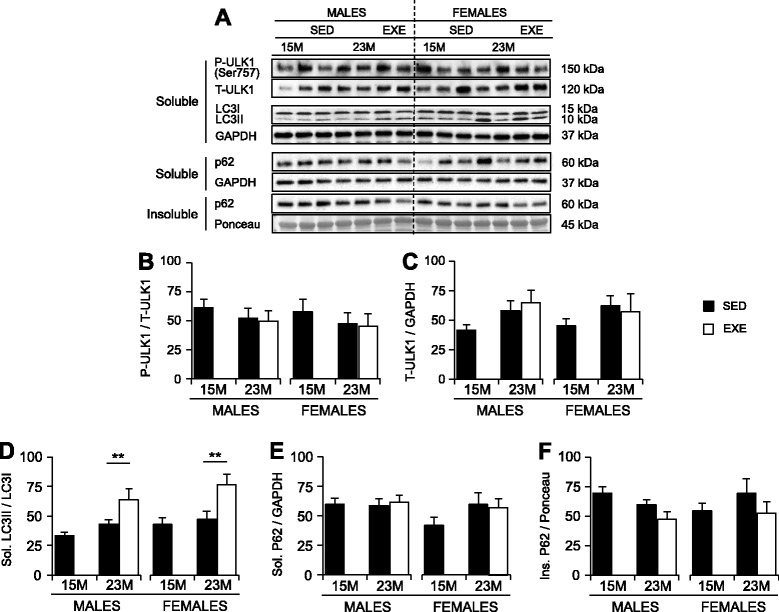
Markers of autophagy in the quadriceps muscles of 15-month SED, 23-month SED, and 23-month RWE mice, of both sexes. P-ULK1(Ser757) was quantified relative to t-ULK1 (**a**, **b**), and t-ULK1 to the loading control GAPDH (**a**, **c**). Ratios of LC3II/LC3I were detected in the 1% NP40 soluble protein fraction, with GAPDH displayed to demonstrate equal loading (**a**, **d**). Protein amounts of p62 were quantified in both the 1% NP40 soluble and insoluble fractions, and standardized relative to GAPDH and Ponceau S (stained band between 50 and 37 kDa), respectively (**a**, **e**, **f**). Data were analyzed by ANOVA, using age and sex and sex and activity as variables. Data are mean ± SEM. *Asterisk* denotes significance at *P < 0.05; **P < 0.01; ***P < 0.001. For each age group, *N* = 6–10 mice/group. Y-axes represent arbitrary units

Phosphorylated amounts of ULK1 (p-ULK1) standardized to t-ULK1, and t-ULK1 standardized to GAPDH, were unaffected by age and were similar between SED males and females (Fig. 6a–c). No age- or sex-specific changes to LC3 lipidation (or the ratio between LC3II/LC3I) (Fig. 6a, d) were observed in the 1% NP40 soluble protein fraction or for p62 in either the 1% NP40 soluble or insoluble protein fractions (Fig. 6a, e, f).

Total and phosphorylated amounts of ULK1 were not influenced by RWE and remained at levels comparable to 23-month-old SED mice in both sexes (Fig. 6a–c). RWE resulted in an increase in LC3 lipidation in the 1% NP40 soluble protein fraction; where the LC3II/I ratio was elevated by 48% in male and 62% in female quadriceps, compared with 23-month-old SED controls (*P* = 0.001; Fig. 6a, d). No significant changes to p62 protein amounts were detected in either the 1% NP40 soluble or insoluble protein fraction in either sex, and p62 was unaffected by RWE when compared to SED 23-month-old controls (Fig. 6a, e, f). However, the amounts of insoluble p62 protein were significantly lower in 23-month EXE males compared with 15-month SED males (Fig. 6a, f; *P* < 0.03).

### Markers of proteasomal degradation

In SED mice, sex had an effect on age-related changes in *Murf1* mRNA expression in the quadriceps muscle, with *Murf1* mRNA increasing (by 52%) in males and decreasing (by 72%) in females, between 15 and 23 months of age in SED mice (Additional file 1: Figure S1A; *P* < 0.05). *Murf1* was not significantly influenced by RWE (Additional file 1: Figure S1A). *Atrogin-1* mRNA expression was not affected by age, sex, or exercise (Additional file 1: Figure S1B).

### Markers of protein synthesis

Levels of phosphorylated and total proteins for AKT, S6K1, and rpS6 were measured in the quadriceps muscles of middle-aged and old mice, to determine levels of anabolic signaling. The quadriceps was chosen because sarcopenia was evident by 23 months of age in both sexes, and this loss of muscle mass was attenuated by RWE (Table 1).

**Fig. 7:**
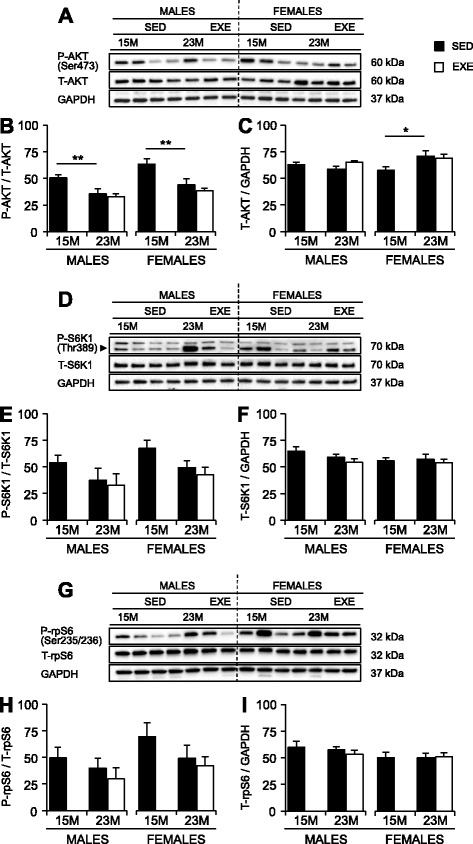
Phosphorylated (p-) and total (t-) protein amounts for AKT, S6K1, and rpS6 in the quadriceps muscles of 15-months SED, 23-month SED, and 23-month RWE mice, of both sexes. p-AKT(Ser473) (**a**, **b**), p-S6K1(Thr389) (**d**, **e**), and p-rpS6(Ser235/236) (**g**, **h**) were quantified relative to their respective total protein amounts. T-AKT (**a**, **c**), t-S6K1 (**d**, **f**), and t-rpS6 (**g**, **i**) were quantified relative to the loading control, GAPDH. Data were analyzed by ANOVA, using age and sex and sex and activity as variables. Data are mean ± SEM. *Asterisk* denotes significance at *P < 0.05; **P < 0.01; ***P < 0.001. For each age group, *N* = 6–10 mice/group. Y-axes represent arbitrary units

In SED mice, the levels of phosphorylated AKT(Ser473) (p-AKT) standardized to total AKT (t-AKT) (Fig. 7a, b) decreased between 15 and 23 months in the muscles of male and female mice (30 and 31%, respectively; *P* = 0.002; Fig. 7a, b). In male mice, t-AKT standardized to GAPDH was similar between 15 and 23 months, although increased by 23% in old SED females (*P* < 0.05; Fig. 7a, c). Overall, the amounts of p-S6K1 standardized to t-S6K1 tended to decrease between 15 and 23 months, with no effect of sex (*P* = 0.06; Fig. 7d, e). There were no significant age- or sex-associated changes to p-rpS6 (Ser235/236) standardized to t-rpS6 (Fig. 7g, h) or to t-S6K1 and t-rpS6 standardized to GAPDH (Fig. 7d, f and g, i).

Exercise had no overall effects on phosphorylated or total amounts of AKT, S6K1, or rpS6, relative to the SED 23-month-old muscles of both sexes.

### Denervation markers

Our previous study in female C57BL/6J mice aged 3–27/29 months [48] and another study in male Sprague Dawley (SD) rats aged 6–27 months [49] identified age-associated changes in expression of genes related to denervation of NMJs. In the present study, we quantified mRNA in the quadriceps muscles for a range of genes: *Gadd45α*, *Runx1*, *Chrng*, *Chrnd*, *Musk*, *and Myog.*

Age increased the overall expression level of *Gadd45α* in the quadriceps although, due to high variation, no significant differences were observed separately for SED males or females (Additional file 2: Figure S2A; main effect *P* < 0.03). *Runx1* expression was increased only in old SED females at 23 months compared with 15 months (Additional file 2: Figure S2B; *P* < 0.05). The levels of mRNAs for acetylcholine receptor subunits in the skeletal muscle were differentially affected by age: while *Chrnd* expression remained unchanged between 15 and 23 months in both SED males and females, *Chrng* expression decreased between 15 and 23 months in males and increased in females (Additional file 2: Figure S2C, D; *P* < 0.05). *Musk* expression was overall higher in SED males compared with females and was not affected by age (Additional file 2: Figure S2E; *P* < 0.05). *Myog* significantly decreased in SED male muscles between 15 and 23 months (*P* < 0.05), in contrast to females where levels remained stable (Additional file 2: Figure S2F).

RWE had variable effects on the expression of both *Gadd45α* and *Runx1*: in males, there was a 60% decrease in *Gadd45α* expression relative to SED 23-month mice (Additional file 2: Figure S2A; *P* < 0.05). A 40% reduction was also observed in exercised females at 23 months, relative to SED controls, but this was not statistically significant due to high variation among individual mice. Exercise reduced *Runx1* mRNA expression by 65% in females, compared with SED controls at 23 months (Additional file 2: Figure S2B; *P* < 0.05), whereas there was no effect of exercise on *Runx1* expression in males. RWE had no significant effect on *Chrng*, *Chrnd*, or *Musk* mRNA levels relative to 23-month SED controls in either sex (Additional file 2: Figure S2C–E). Similarly, *Myog* expression levels in males following RWE was similar to 23-month-old SED levels, although *Myog* in exercising female mice tended to increase relative to age-matched controls (Additional file 2: Figure S2F; *P* < 0.06).

## Discussion

This is the first study to assess the benefits of long-term voluntary RWE on sarcopenia in aging male and female mice. We show that RWE (from 15 to 23 months) in both male and female C57BL/6J mice significantly prevented total body weight gain and abdominal fat mass, caused cardiac hypertrophy (greater in females), and prevented sarcopenia in both sexes. Specifically, weights of the exercised quadriceps, gastrocnemius, and EDL muscles at 23 months were maintained at values similar to 15 months, while mass of the soleus muscles increased. At the molecular level, RWE increased markers of mitochondrial density and activity, and increased LC3II/I ratios, a marker of autophagy. The impact and significance of RWE, age, and sex on this range of parameters are discussed below.

### Phenotype: running distance, speed, and external work

The distances run by male and female mice on loaded wheels from 15 to 23 months of age were equivalent to our previous report [50] and, in some cases, exceeded those reported by others for unloaded (free spinning) wheels [21]. Mice in the present study had typical circadian running patterns, characterized by two peaks of activity: one 2–3 h prior to lights on and another 2–3 h prior to lights out, which is a characteristic of young, rather than old C57BL/6J mice where activity is defined by only one main peak of activity [24]. Given the voluntary nature of wheel running, distances run by mice in the present study were inversely proportional to the wheel load and decreased from an average of 4.6 and 5.9 km per day for males and females, respectively, on an unloaded wheel, to an average of 2 and 1.4 km per day at 6 g resistance (W21–34). While C57BL/6J mice are less avid wheel runners than other strains [51], we have previously shown that male C57BL/6J mice (aged ~25–27 months) are capable of running approximately 3 km per night on an unloaded wheel and 0.5 km with 4 g of resistance [24].

To date, loaded wheel running protocols have only been tested in male mice and rats [33, 52, 53]. However, notable sex-specific differences have been reported on unloaded wheels in C57BL/6J mice. For example, C57BL/6J females are reported to run on average 40% further than males, at greater speeds, and for longer at both young (4 months) and middle ages (12–15 months) [54]. Although recent studies in C57BL/6J mice demonstrate that differences in running activity are more prominent between males and females at younger ages (2 months), due to higher achieved velocities. Interestingly, these sex differences become negligible by 33–40 weeks of age (~7.5–9 months) and are not evident at 24 months [55]. Age-related hormonal changes in female mice may be responsible for equalized running activity between the sexes at older ages. Indeed, there is evidence that exercise performance may depend on the presence of estrogen, since wheel running is reduced in ovariectomized mice and rats [56, 57].

Both male and female mice maintained levels of work throughout our study from mid-life (15 months) to old age (23 months). Konhilas et al. (2005) showed that young (aged 3–4 months) male C57BL/6J mice can tolerate increasing resistance on the running wheel up to 7 g (up to 25% of total body weight) before external work is negatively affected. The long-term impact of increasing resistance and its effect on the level of work has not been addressed in aging mice, prior to the present study. While our resistance protocol is relatively moderate (gradual increase up to 6 g over 21 weeks), our data support both a gain and retention of muscle function into old age for both sexes (as demonstrated by the maintenance of work at 24 months, which is comparable to 15-month baseline levels). The ability to sustain workload and overall aerobic exercise capacity can be influenced by many physiological factors, such as muscle strength and cardiopulmonary fitness, including cardiac output, maximal oxygen uptake, and peripheral oxygen capacity (among others) [58, 59]. While such physiological measurements were outside the scope of this paper, we did observe increased heart weights after RWE in both sexes, with female mice exhibiting a greater increase than males. Increased heart mass (greater in females) has been previously reported in response to unloaded voluntary wheel exercise comparing both sexes [54, 60] and forced exercise regimes like swimming where only females were analyzed [61]. While these studies suggest that exercise performance is not dependent on the extent of cardiac hypertrophy, it is likely to support an increase in exercise performance and aerobic capacity, at least in rodents.

### Muscle mass

Similar to reports in elderly men and women (reviewed in [4]), we show that sarcopenia is more severe in SED male compared with female mice, and that the rate of atrophy differs significantly between the muscle groups. Of the SED muscles analyzed at 23 months (compared with 15 months), the weight of the quadriceps, gastrocnemius, soleus, and TA decreased (up to 16%) in males, whereas reduced muscle mass was observed only for the quadriceps muscle (by 13%) in females. In mice, sarcopenia may vary significantly between the muscle groups and can be influenced by muscle location and biomechanics and usage [62].

In response to the sustained RWE from 15 months of age, the soleus showed marked hypertrophy, with increased CSA evident in all old mice. This accords with our previous findings that the soleus exhibits the greatest hypertrophic response to exercise in both young and old mice [24]: specifically, we showed that 10 weeks of RWE (up to 4 g) late in life (from ~25–27 months) induce hypertrophy of the soleus muscle (up to 18%) in very old, sarcopenic male mice [24]. In general, hypertrophy (increased myofiber CSA) has previously been noted in both forelimb and hindlimb muscles in response to loaded wheel-running protocols (of both low and high resistance) in young mice and rats (aged 5–25 weeks) [24, 33, 52]. Furthermore, RWE can induce hypertrophy in a range of muscles in young mice and rats [24, 52], and to a greater extent than that observed in the older muscles [24]. We have previously shown that life-long exercise (from 4 months) on unloaded wheels prevents the loss of quadriceps mass (evident in sedentary mice at 28 months), with variable effects on the other hindlimb muscles [50]. The present study shows that sustained resistance wheel exercise from mid-life (15 months, before the onset of sarcopenia) is sufficient to prevent sarcopenia and maintain skeletal muscle mass into old age.

### Mitochondrial content and oxidative capacity

While a variety of mitochondrial abnormalities become more common in the sarcopenic muscles [63], in the present study, no changes to CS activity were observed (used as an indicator of mitochondrial density) between middle (15 months) and old (23 months) age groups. Our results accord with those of Jackson et al. (2011) who reported unchanged CS activity in the gastrocnemius muscles between middle and old age (18 and 28 months) C57BL/6J mice [64]. This lack of age-related change is further supported by reports of similar levels of CS activity in the quadriceps muscles of young adult (aged 3–5 months), adult/middle-age (10–12 months), and old (20–22 months) BALB/c mice [65]. We also observed only modest (overall) decreases to whole muscle NADH-TR staining intensities and thus mitochondrial oxidative enzyme activity in the quadriceps between 15 and 23 months of age. Thus, our data indicate limited changes to mitochondria (with respect to these parameters) in the quadriceps muscles between middle and old ages in C57BL/6J mice.

RWE initiated from mid-life increased CS activity in the quadriceps and gastrocnemius muscles of all mice. Greater NADH-TR staining intensities in exercised quadriceps also demonstrate an enhanced capacity for oxidative metabolism in old muscle. Data for our exercised muscle accords with previous reports in young rodents (aged 11–28 weeks), where prolonged voluntary wheel running (unloaded) for 4 to 20 weeks increased key markers of mitochondrial biogenesis, oxidative phosphorylation, and intramuscular mitochondrial content [27, 28]. Skeletal muscles rely on mitochondria to meet the higher demand for ATP generated during sustained contractile activity, and both mitochondrial content and function greatly impact muscle performance [63]. Many studies in middle-aged and old rodents demonstrate that mice [66–68] and rats [69–71] of both sexes (aged 13–32 months) maintain the ability to increase muscle mitochondrial enzymes through forced or voluntary exercise, despite older mice running lower maximum daily distances (old <1 km and young >10 km per day) [66].

### Regulation of protein degradation: autophagy and the proteasome

Maintenance of healthy muscle mass and function also requires removal of damaged proteins and organelles by protein degradation pathways that include autophagy and the ubiquitin proteasome pathway. To evaluate autophagy, ULK1 and p62 protein levels were quantified in combination with the LC3II/LC3I ratio [47, 72–75]. Insufficient autophagy can result in the accumulation of damaged and aggregated proteins, which are poorly soluble in ionic detergents (NP40 and Triton X-100) [76]. We have previously shown that p62 accumulates in the ionic detergent insoluble protein fraction between 4 and 15 months of age in male C57BL/6J muscle and remains elevated up until 24 months [47]. In accordance with our previous findings in male C57BL/6J mice, we observed no changes to the markers of autophagy, ULK1(Ser757), p62, or LC3II/I, between 15 and 23 months of age in either sex [47]. ULK1 is activated under low cellular nutrient states to initiate autophagosome formation [42, 77]. Since ULK1(Ser757) is an mTORC1-specific phosphorylation site [41, 43, 44], the lack of ULK1(Ser757) regulation (and other downstream autophagy markers) with age is also consistent with the observation of unchanged mTORC1 activity, as measured by phosphorylation of its other downstream substrate S6K1(Thr389). AKT can negatively regulate autophagy via the inactivation of the FoxO3 transcription factor [78, 79], and under conditions of muscle atrophy FoxO3 induces transcription of the autophagy-related genes including LC3 and p62 [80]. We did not measure the activity of FoxO3 nor mRNA levels for LC3 and p62; thus, we can only comment that lower AKT(473) phosphorylation in 23-month-old male and female SED mice, compared with 15 months, was not associated with changes in p62 protein levels or LC3 lipidation.

By contrast, elevated ratios of LC3II/I (indicative of LC3 lipidation) following RWE suggest increased levels of basal autophagy, although no changes to p62 protein amounts were observed in the present study. While it has been reported that exercise regimes can increase markers of autophagy in the skeletal muscles [27, 28, 72], changes to the LC3II/I ratio and p62 amounts can vary between protocols, possibly due to differences in exercise intensity, duration, and frequency. For example, in young male C57BL/6J mice, a trend for increased LC3 lipidation in combination with decreased p62 protein amounts have been observed following 4 weeks of unloaded wheel running in the plantaris muscles of 15-week-old mice [28]. A trend for increased LC3 lipidation was also observed in the plantaris muscles of female SD rats (aged 7 months) following 20 weeks of unloaded wheel running; however, p62 protein decreased [27]. One study using tail-weighted resistance exercise over 9 weeks (3 days/week) reduced both LC3II/I ratios and p62 protein amounts in the gastrocnemius muscles of aged male SD rats (18–20 months) [81], whereas no changes to either protein were observed in the same muscles of younger adult male SD rats (aged 10 months) following 36 weeks of treadmill running for 30 min/day, 4–5 days/week (starting from 5 weeks of age) [82]. Interestingly, in the present study, an increase in the LC3II/I ratio following RWE occurred independently of changes to AKT(Ser473) or ULK1(Ser757) phosphorylation.

To evaluate the proteasomal degradation pathway, we examined transcript levels of E3 ubiquitin ligases *Murf1* and *Atrogin-1* [83] in SED and RWE mice. MuRF1 is a muscle-specific ubiquitin ligase that facilitates degradation of thick filaments during muscle atrophy [84, 85]. The biological role of Atrogin-1 is less well understood; however, it is proposed that Atrogin-1 controls protein synthesis by degrading eIF3f and thus suppressing S6K1 activation via mTORC1 [86]. While *Atrogin-1* remains relatively stable in the quadriceps muscles of freely fed male and female C57BL/6J mice between 3–4 and 24 months of age [47, 48] and the gastrocnemius of male SD rats between 6 and 27 months [49], there is no consensus with regard to *Murf1* expression, at least in rodents [47–49, 87]. Differences in the published reports may be due to differences in genders, muscles, and ages used or even differences in husbandries. Importantly, because *Murf1* amounts may rise and fall during muscle atrophy [88] and sarcopenia [48], a detailed aging time-course comparison is required to establish if and when this ligase is induced in the aging muscles (reviewed in [89]).

In male SD rat gastrocnemius muscles, *Murf1* transcripts are already elevated at 12 months compared with 6 months and continue to increase up to 27 months [49], whereas decreased *Murf1* transcripts were reported in the gastrocnemius muscles of female SD rats between 4–12 and 30 months [87]. We previously examined MuRF1 mRNA and protein levels in the quadriceps muscles of female C57BL/6J mice aged 3, 15, 24, 27, and 29 months [48] and male mice aged 4, 15, 18, 22, and 24 months [47]. In females, *Murf1* mRNA transiently increased between 15 and 24 months, with increased association of this E3 ligase with the myosin-enriched protein fraction [48]. In males, we observed increased association of MuRF1 protein with the myosin-enriched protein fraction between 18 and 24 months with no changes in *Murf1* mRNA across five ages [47].

The present study compared *Murf1* mRNA expression between young and old male and female mice in the same experiment. In SED mice, sex affected age-related changes in *Murf1* expression levels, with mRNA levels increased in males but decreased in female muscles between 15 and 23 months: this accords with the sex differences in aging rats (discussed above). The present experimental design precludes us from concluding that sarcopenia in female quadriceps muscles occurred without upregulation of *Murf1*, because expression of *Murf1* mRNA is thought to occur in waves in murine muscles undergoing sarcopenia [48]. Phosphorylated AKT is a major suppressor of *Murf1* expression, achieved by suppression of FoxO transcription factors [90–92]. In our study, pAKT was reduced in both SED male and female quadriceps at 23 months, compared with 15 months, although *Murf1* was differentially regulated. A disconnection between the pAKT and *Murf1* expression is not an unusual occurrence in vivo and has been observed in sarcopenic muscles of female C57BL/6J mice [48] and in surgically denervated muscles [93–95].

RWE did not affect *Murf1* and *Atrogin-1* expression levels: these remained similar to age-matched SED controls for all mice. Our results accord with studies in both young (aged 25/26 years) and old (76–86 years) human muscles, where both resistance and endurance exercise ranging in duration from 5 to 21 weeks had no effect on *Murf1* or *Atrogin-1* expression [32, 96–98]. Similarly, *Murf1* and *Atrogin-1* mRNA amounts remained stable between elderly men who only performed routine daily activities (SED; 65–74 years) and senior sportsmen engaged in life-long sporting activities at least three times weekly (aged 65–79 years) [99]. Few studies have described the regulation of *Murf1* and *Atrogin-1* following chronic exercise in rodents. Cunha et al. (2012) demonstrated that *Murf1* expression is differentially affected by exercise duration and increased following 8, but not 2, weeks of treadmill running (5 days/week of progressing increased duration and speed) in the plantaris muscles of adult male C57BL/6J mice (aged 7 months). The same study found no changes to *Atrogin-1* expression [100]. Protein amounts of MuRF1 and Atrogin-1 (that were not quantified in the present study) were recently shown to remain stable after 6 weeks of treadmill activity (5 days/week) when measured in the gastrocnemius muscles of male Wistar Kyoto rats at 11 weeks of age [101]. Thus, it appears that key markers of proteasomal degradation are relatively unaffected in both human and rodent muscles in response to chronic forms of exercise training.

### Regulation of protein synthesis (AKT-mTORC1) pathway

The mTORC1 signaling pathway is a key regulator of protein homeostasis in the skeletal muscle, and activation of mTORC1, either downstream of AKT or directly by nutrients promotes protein synthesis by phosphorylating two major targets; S6K1 (along with downstream rpS6) and 4E-BP1 [102–104]. In the present study, pAKT(Ser473) decreased between 15 and 23 months of age in SED mice of both sexes, with no differences seen for S6K1(Thr389) and rpS6(Ser235/236). We have previously characterized levels of pAKT(Ser473), pS6K1(Thr389), and p-rpS6(Ser235/236) in the quadriceps muscles of freely fed female [48] and male [47] C57BL/6J mice across a variety of ages. These studies showed similar levels of pAKT(Ser473) in the skeletal muscles of freely fed young, middle-aged, and old female and male C57BL/6J mice [47, 48]. A significant increase was observed for p-rpS6(Ser235/236) in freely fed female muscles at 24 months compared with 15 months [48], whereas in male muscles, p-rpS6(Ser235/236) was elevated at 22 months compared with 15 months, with no difference observed between 15 and 24 months [47, 48].

Additional studies in fasted mice (young adult compared with old) have established baseline levels of mTORC1 signaling [47, 103], which may be more reliable, than freely fed levels, as this pathway is significantly affected by both nutrients and insulin that increase during feeding [34, 105]. In aged mouse muscles, several published reports agree that in the fasted state (at least in males), aging results in increased activity of AKT and mTORC1 signaling [47, 103]. Since muscles in the present study were collected from freely fed mice, we cannot reliably determine whether pAKT(S473) or mTORC1 signaling were affected by aging.

In the present study after RWE (that prevented sarcopenia in the quadriceps), we did not observe increased levels of pAKT, pS6K1, or p-rpS6 for either sex. This may be due to the sustained duration of RWE over 34 weeks, since the phosphorylation of AKT and both S6K1 and rpS6 occurs rapidly following a single, acute bout of exercise, and progressively decreases over time [106–108]. Given our experimental design, we cannot determine if the RWE protocol resulted in an earlier, acute transient increase of AKT phosphorylation or activity of the mTORC1 pathway. Our data accord with studies in elderly humans that investigated the chronic effects of exercise on intramuscular anabolic signaling, where similar levels of pAKT(Ser473) [109], pS6K1(Thr389) [109, 110], p-rpS6(Ser235/236) [109], and p4E-BP1(Thr37/46) [110] were observed in the vastus lateralis muscles of young and old men (aged 18–41 and 60–86 years) after 12 weeks of resistance training, relative to age-matched SED controls.

### Denervation markers

Functional denervation of myofibers, combined with physical changes at the NMJ, has been reported in aged mice [7, 25, 48, 111], rats [49], and humans [7, 112]. In addition, a novel time course analysis of aging peripheral nerves of male and female C57BL/6J mice (where sarcopenia was evident) demonstrated increasing levels of many proteins in old sciatic nerves [113]. This recent study, combined with a recent comprehensive study in aging male rats that included transcriptome analysis of sciatic and radial nerves, and gastrocnemius and triceps brachii muscles, combined with imaging of NMJ in EDL and the biceps brachii [7], strongly emphasized age-related changes to the neuromuscular system during sarcopenia. However, the extent to which neuronal changes contribute to, or are a consequence of, sarcopenia remains unclear.

An important finding in the present study was that only some, but not all, of the denervation biomarkers (*Gadd45α*, *Runx1*, *Chrnd*, *Chrng*, *Musk*, *and Myog)* were regulated by age, and a significant sex-specific variability was observed. These genes have been used as markers of myofiber denervation because their expression increases in surgically denervated muscles [114–120]. Importantly, increased expression of these genes also has been reported in old rodent skeletal muscle and is proposed to be associated with disturbances to the NMJ and myofiber denervation [7, 48, 49, 116, 121]. Increased mRNA levels for *Chrng*, *Chrnd*, *Musk*, and *Myog* are reported for old male SD rat gastrocnemius muscles from as early as 12 months of age (although for most at 18–21 months), with progressive increases up to 27 months age [49]. A recent report by Aare et al*.* (2016) also shows that *Chrng*, *Musk* , and *Runx1* mRNA amounts are upregulated in the vastus lateralis muscles of male F344BN rats between 8 and 35–36 months of age [121]. We have earlier demonstrated a significant increase in *Chrng*, *Chrnd*, *Myog*, *Gadd45α*, and *Runx1* expression in the quadriceps muscles of female C57BL/6J mice between 15 and 24 months although expression of these genes became highly variable at later ages (27–29 months) [48]. Overall, the above studies imply some relationship between increased mRNA levels of the selected denervation biomarkers within the skeletal muscle and neuromuscular health. The precise consequences of all these changes on muscle contraction can be hard to determine.

The present study examined only mRNA levels for these denervation biomarkers. Although quadriceps muscle mass was reduced in old mice of both sexes, only old females showed a significant increase in some (*Runx1* and *Chrng*) of the examined markers of myofiber denervation: the *Gadd45α* mRNA increase was modest and had a main effect of age only. A significant increase of *Runx1* and *Chrng* mRNA in old female quadriceps muscles accords with our previous report for old female C57BL/6J mice [48]. However, previously reported significant increases in *Gadd45α*, *Chrnd*, and *Myog* in the 24-month-old female quadriceps muscles [48] were not seen in the present study that analyzed mice aged 23 months: younger age may have been one reason for such discrepancies between studies. In SED male quadriceps, the absence of striking age-related increases in mRNA levels of denervation markers suggests that myofiber denervation was not pronounced in these muscles, although sarcopenia had occurred. The present study indicates that age-related upregulation of genes for denervation biomarkers can be influenced by gender and may be highly variable between studies.

RWE suppressed age-related increases in expression of *Gadd45α* and *Runx1* in old male and female quadriceps muscles, respectively, and this was associated with amelioration of sarcopenia. *Gadd45α* expression increases in situations of skeletal muscle atrophy (due to starvation, denervation, disuse, and aging) and promotes loss of muscle mass possibly via forming a complex with MEKK4 kinase and increasing its activity [115, 122, 123]. Muscle-specific ATF4 knock-out mice that have a reduced capacity to induce *Gadd45α* mRNA in response to stress undergo less atrophy in response to fasting or muscle immobilization [122]. In the present study, reduced *Gadd45α* expression following RWE may contribute to the beneficial effect of exercise on sarcopenia in these mice.

Conversely, increased *Runx1* expression in surgically denervated muscle [119] is proposed to protect myofibers from severe atrophy [118]. The suppressed expression of *Runx1* in female muscle by RWE in the present study accords with observations in humans, where long-term (5-month, 3 days/week) resistance training in 65–79-year-old men and women increased vastus lateralis muscle strength with a concurrent decrease in *Runx1* and *Chrng* expression relative to baseline (or pre-training) levels [124].

### Wider implications of aging and RWE

This study focussed on the hindlimb muscles in aging mice subjected to RWE. It is increasingly being recognized that exercise-induced muscle contractions and the loading of muscle fibers releases many factors into the circulation that affect a wide range of tissues and systemic metabolism (reviewed in [125]), with presumably beneficial feedback from (at least some of) these other tissues to the aging muscles. This wider feedback, with effects on muscle fibers, may include changes to the vasculature, local extracellular matrix composition, inflammation, and innervation. While the role of the nervous system in sarcopenia and response to RWE (Krishnan et al. manuscript under review) is attracting increasing attention, the impact of exercise on other cell types and tissues and consequences for sarcopenia remain to be explored. A deeper understanding of the benefits of exercise and complexity of systemic interactions has potential therapeutic consequences for many tissues, including the healthy maintenance of old muscle mass and function.

## Conclusions

Overall, our data show that the introduction of resistance wheel running from middle age was effective in preventing sarcopenia in the hindlimb muscles of both male and female mice. The maintenance of muscle mass into old age was accompanied by striking changes to morphological and molecular parameters of the muscles, including myofiber size and type, with some increased markers of mitochondrial and autophagic activity. Since exercising muscles produce many factors with systemic effects, it is possible that other tissues may subsequently feedback and contributes (indirectly) to the prevention of sarcopenia, by exercise. This study shows that aging mice of both sexes have a good capacity for such resistance exercise and that this exercise helps to maintain healthy old muscles.

## Additional files

Additional file 1: Figure S1.
*Murf1* (A) and *Atrogin-1* (B) mRNA in quadriceps muscles of 15-month SED, 23-month SED, and 23-month RWE mice, of both sexes. Gene expression in the quadriceps muscles was normalized to the geometric mean of *Hprt* and *Ppia* expression values. Data were analyzed by ANOVA, using age and sex, and sex and activity as variables. Data are mean ± SEM. Asterisk (*) denotes significance at **P* < 0.05; ***P* < 0.01; ****P* < 0.001. For each age group, *N* = 5–9 mice/group. Y-axes represent arbitrary units.
Additional file 2: Figure S2.
*Gadd45α* (A), *Runx1* (B), *Chrnd* (C), *Chrng* (D), *Musk* (E), and *Myog* (F) mRNA in the quadriceps muscles of 15-month SED, 23-month SED, and 23-month RWE mice, of both sexes. Gene expression in the quadriceps muscles was normalized to the geometric mean of *Hprt* and *Ppia* expression values. Data were analyzed by ANOVA, using age and sex, and sex and activity as variables. Data are mean ± SEM. Asterisk (*) denotes significance at **P* < 0.05; ***P* < 0.01; ****P* < 0.001. For each age group, *N* = 5–9 mice/group. Y-axes represent arbitrary units.
