# Erratum to: Voluntary resistance wheel exercise from mid-life prevents sarcopenia and increases markers of mitochondrial function and autophagy in muscles of old male and female C57BL/6J mice

**DOI:** 10.1186/s13395-017-0120-3

**Published:** 2017-02-15

**Authors:** Zoe White, Jessica Terrill, Robert B. White, Christopher McMahon, Phillip Sheard, Miranda D. Grounds, Tea Shavlakadze

**Affiliations:** 1 School of Anatomy, Physiology and Human Biology, The University of Western Australia (UWA), 35 Stirling Highway, Crawley, WA 6009 Australia; 2 Centre for Cell Therapy and Regenerative Medicine, School of Medicine and Pharmacology, UWA and Harry Perkins Institute of Medical Research, Crawley, 6009 WA Australia; 3 School of Chemistry and Biochemistry, UWA, Crawley, 6009 WA Australia; 4 Developmental Biology Group, AgResearch Ltd, Hamilton, 3214 New Zealand; 5 Department of Physiology, University of Otago, Dunedin, 9010 New Zealand

## Erratum

Following publication of the original article [1] it was brought to our attention that there was a problem with the merging of the lines in Figs. 6 and 7. These figures show western blot images and each image used to have lines indicating separate groups. During production these lines merged into one single line and now the separate groups cannot be identified. Please see below for the corrected images:

**Fig. 6:**
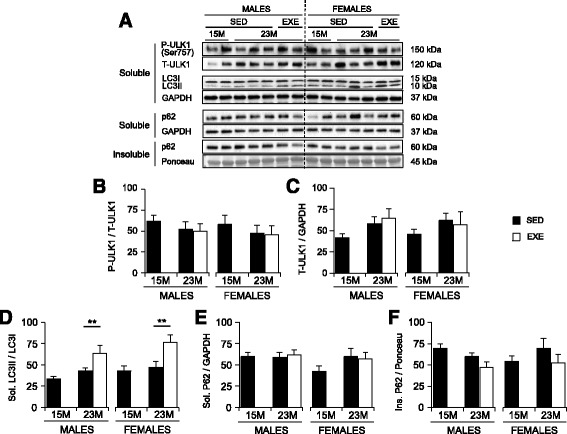
Markers of autophagy in the quadriceps muscles of 15-month SED, 23-month SED, and 23-month RWE mice, of both sexes. P-ULK1(Ser757) was quantified relative to t-ULK1 (**a**, **b**), and t-ULK1 to the loading control GAPDH (**a**, **c**). Ratios of LC3II/LC3I were detected in the 1% NP40 soluble protein fraction, with GAPDH displayed to demonstrate equal loading (**a**, **d**). Protein amounts of p62 were quantified in both the 1% NP40 soluble and insoluble fractions, and standardized relative to GAPDH and Ponceau S (stained band between 50 and 37 kDa), respectively (**a**, **e**, **f**). Data were analyzed by ANOVA, using age and sex and sex and activity as variables. Data are mean ± SEM. *Asterisk* denotes significance at *P < 0.05; **P < 0.01; ***P < 0.001. For each age group, *N* = 6–10 mice/group. Y-axes represent arbitrary units

**Fig. 7:**
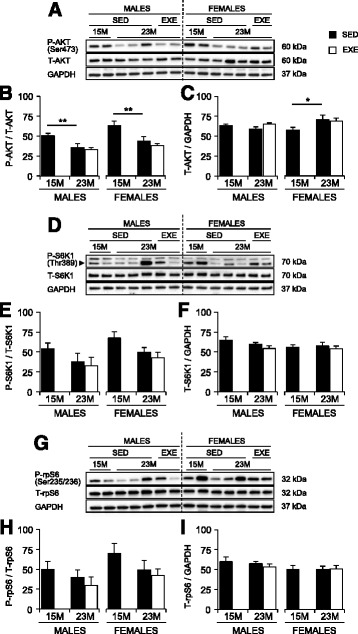
Phosphorylated (p-) and total (t-) protein amounts for AKT, S6K1, and rpS6 in the quadriceps muscles of 15-months SED, 23-month SED, and 23-month RWE mice, of both sexes. p-AKT(Ser473) (**a**, **b**), p-S6K1(Thr389) (**d**, **e**), and p-rpS6(Ser235/236) (**g**, **h**) were quantified relative to their respective total protein amounts. T-AKT (**a**, **c**), t-S6K1 (**d**, **f**), and t-rpS6 (**g**, **i**) were quantified relative to the loading control, GAPDH. Data were analyzed by ANOVA, using age and sex and sex and activity as variables. Data are mean ± SEM. *Asterisk* denotes significance at *P < 0.05; **P < 0.01; ***P < 0.001. For each age group, *N* = 6–10 mice/group. Y-axes represent arbitrary units
