# Supplementary figures and images for: Voluntary resistance wheel exercise from mid-life prevents sarcopenia and increases markers of mitochondrial function and autophagy in muscles of old male and female C57BL/6J mice
Source: Skelet Muscle. 2016 Dec 13;6:45. doi: 10.1186/s13395-016-0117-3 (PMC5155391; doi:10.1186/s13395-016-0117-3)

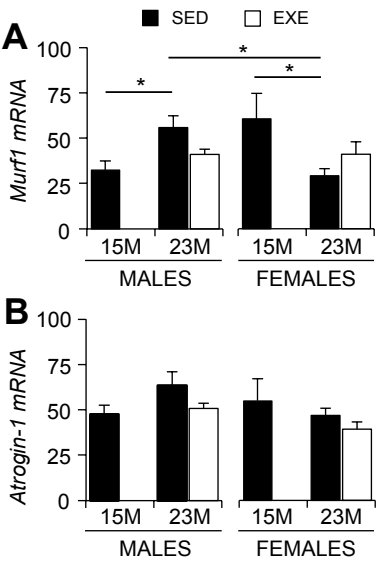

Supplement: Additional file 1: Figure S1. — Murf1 (A) and Atrogin-1 (B) mRNA in quadriceps muscles of 15-month SED, 23-month SED, and 23-month RWE mice, of both sexes. Gene expression in the quadriceps muscles was normalized to the geometric mean of Hprt and Ppia expression values. Data were analyzed by ANOVA, using age and sex, and sex and activity as variables. Data are mean ± SEM. Asterisk (*) denotes significance at *P < 0.05; **P < 0.01; ***P < 0.001. For each age group, N = 5–9 mice/group. Y-axes represent arbitrary units. [file 13395_2016_117_MOESM1_ESM.pdf]

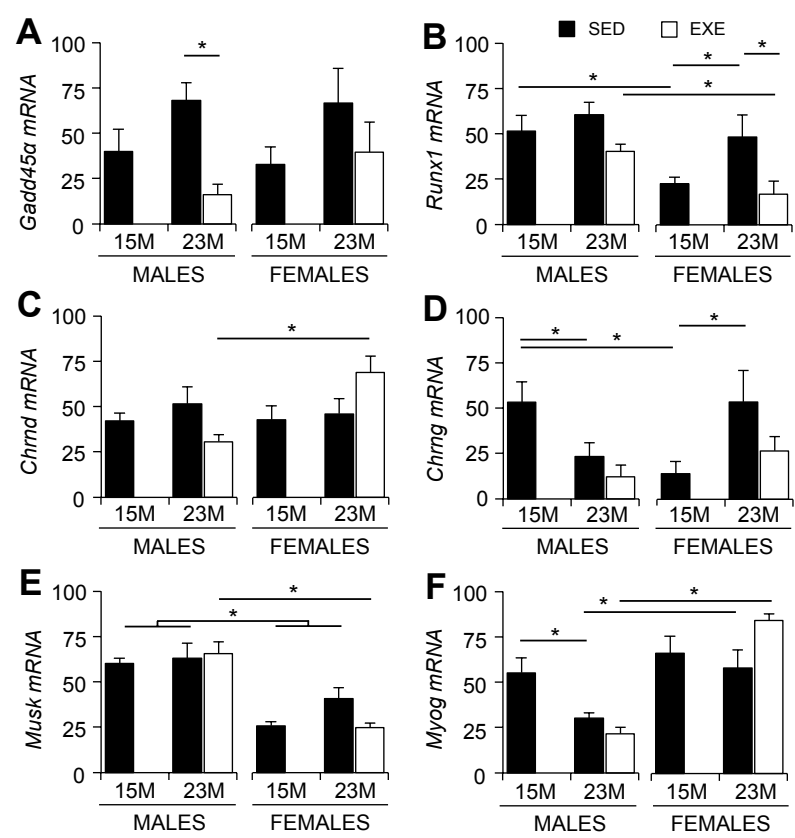

Supplement: Additional file 2: Figure S2. — Gadd45α (A), Runx1 (B), Chrnd (C), Chrng (D), Musk (E), and Myog (F) mRNA in the quadriceps muscles of 15-month SED, 23-month SED, and 23-month RWE mice, of both sexes. Gene expression in the quadriceps muscles was normalized to the geometric mean of Hprt and Ppia expression values. Data were analyzed by ANOVA, using age and sex, and sex and activity as variables. Data are mean ± SEM. Asterisk (*) denotes significance at *P < 0.05; **P < 0.01; ***P < 0.001. For each age group, N = 5–9 mice/group. Y-axes represent arbitrary units. [file 13395_2016_117_MOESM2_ESM.pdf]
